# Impact of defect occupation on conduction in amorphous Ge_2_Sb_2_Te_5_

**DOI:** 10.1038/srep31699

**Published:** 2016-08-16

**Authors:** Matthias Kaes, Martin Salinga

**Affiliations:** 1 I.Physikalisches Institut (IA), RWTH Aachen University, Sommerfeldstrasse 14, 52074 Aachen, Germany

## Abstract

Storage concepts employing the resistance of phase-change memory (PRAM) have matured in recent years. Attempts to model the conduction in the amorphous state of phase-change materials dominating the resistance of PRAM devices commonly invoke a connection to the electronic density-of-states (DoS) of the active material in form of a “distance between trap states *s*”. Here, we point out that *s* depends on the occupation of defects and hence on temperature. To verify this, we numerically study how the occupation in the DoS of Ge_2_Sb_2_Te_5_ is affected by changes of temperature and illumination. Employing a charge-transport model based on the Poole-Frenkel effect, we correlate these changes to the field- and temperature-dependent current-voltage characteristics of lateral devices of amorphous Ge_2_Sb_2_Te_5_, measured in darkness and under illumination. In agreement with our calculations, we find a pronounced temperature-dependence of *s*. As the device-current depends exponentially on the value of *s*, accounting for its temperature-dependence has profound impact on device modeling.

Memory-technology continues to facilitate developments at the forefront of science. For example, the ground-based next-generation astronomical survey ‘square-kilometer array’ (SKA) will produce extreme amounts of data during its extensive mapping of unexplored regions of space-time^1^. For SKA, the yearly amount of raw-data is projected to amount to roughly 22 × 10^9^ terabyte^2^, and hence, deriving information requires a combination of event-filtering, data-mining and appropriate solutions for data-storage. As in other fields of ‘big-data’ such as genomics (see e.g. ref. 3), a memory-related challenge is to meet the required latency for data retrieval/storage at minimum cost^4^. In consequence, these endeavors will benefit from the increasing diversification that characterizes today’s memory-market.

This technological diversity is related to the emergence of non-volatile resistive memories such as conductive-bridge RAM (CBRAM) or phase-change memory (PRAM), loosely grouped as ‘storage-class memory’ (SCM)^5^. In SCMs, the resistance of the memory-element can be read and manipulated by suitable external stimuli (voltage/current pulses) enabling retrieval and storage of information. Their main advantage compared to NOR and NAND flash is that read/erase operations can usually be performed faster and sustained with higher endurance^6^. Last year’s introduction of 3D-XPoint™ memory by Micron/Intel demonstrated that SCMs relying on resistive switching of chalcogenides are technologically mature and commercially viable^7^. Although details of the active material in the memory elements as well as the selector devices remain undisclosed, this recent development puts a spotlight on a whole class of materials that has been studied since the discovery of threshold switching in the 1960s^8^.

Chalcogenides can be switched reversibly between a highly-resistive amorphous (reset) and a highly-conductive crystalline (set) state. While the transition from the set- to the reset-state is usually obtained via melt-quenching, the set-transition is characterized by a breakdown of the resistance that occurs at threshold fields of 20 V/μm to 50 V/μm^9^. This breakdown leads to a substantial increase of current which can then induce crystallization through substantial Joule-heating. The time-scale on which crystallization can occur separates the so-called phase-change materials (PCM) from the ovonic-threshold switching materials (OTS). PCMs such as GeTe can crystallize within a few nanoseconds^10^, whereas the atomic structure of OTS-materials remains unchanged even under significant Joule-heating. However, both kinds of amorphous materials share a prerequisite for resistive switching i.e. a highly non-linear current-voltage characteristic. Despite intensive research, the origins of this non-linearity are still debated today.

Many attempts to explain the non-linearity of subthreshold conduction in OTS-materials have resorted to the Poole-Frenkel (PF) mechanism^11^,^12^,^13^. Although other mechanisms such as field-induced nucleation^14^ and small-polaron hopping^15^ have been proposed to explain the non-linearity in the case of PCMs too, also for these materials the PF-mechanism is commonly invoked to model the field- and temperature-dependence of the device-current in the subthreshold and threshold regime^16^,^17^,^18^,^19^,^20^. In essence, this model describes how the electric field enhances the emission probability of charge-carriers trapped in the Coulomb potential of a defect state in the bandgap.

The enhancement of the emission probability occurs because the energetic barrier for emission is lowered by the electric field. Due to this lowering, the number of charge-carriers emitted into the band and thus the conductivity increases. If the density of sites with Coulomb potentials is sufficiently high, their overlap may lead to an additional barrier-lowering^11^,^21^,^22^. For example, the defect density in amorphous GeTe, e.g. *N*_*D*_ ~ 4 × 10^20^ 1/cm^3^ in the shallow-defect^23^, corresponds to average distances as low as 1.4 nm and thus could play a role in field-enhanced emission. Consequently, conduction models invoke a parameter describing this defect density - the inter-trap distance *s*^16^,^24^.

This parameter is crucial for modeling device-characteristics as the subthreshold current depends on it in an exponential way. This becomes especially important if the device-state is read at high fields as in the non-resistance-based metrics proposed recently^25^,^26^,^27^. In all modeling approaches so far, e.g. refs 16, 28 and 29, this inter-trap distance describes the electronic states in a rather unspecified manner i.e. without drawing a connection to a specific defect. Moreover, it is treated as a constant. Especially the latter is conceptually puzzling as the Coulomb interaction of a trapped charge-carrier with a neighboring defect must depend on the charge-state of that defect. The charge-state of a defect in turn should depend on its state of occupation. Thus, the fundamental temperature-dependence of the occupation function should affect the inter-trap distance.

Here, we ask the following questions a) Does the inter-trap distance *s* depend on the occupation of defect-states and thus on temperature? and b) Is it possible to relate the inter-trap distance to a specific feature in the electronic density of states (DoS)?

Elucidating these questions will be of fundamental and practical value. The understanding of the relation between the DoS and the inter-trap distance constitutes a crucial step towards tailoring the current-voltage characteristics of PRAM and OTS devices by material design. Clearly, the temperature-dependence of the inter-trap distance - hitherto not accounted for^29^,^30^ - must be consequential in a physically correct simulation of switching in such devices.

To investigate the connection between the inter-trap distance and the occupation of defects, we deliberately change the occupation of defects in the prototype PCM, Ge_2_Sb_2_Te_5_, by illuminating our devices with light. We assess the impact of illumination and temperature by measuring the electrical transport in micro-scale devices of amorphous Ge_2_Sb_2_Te_5_ (GST) in darkness and under illumination. To span a wide temperature range and at the same time prevent (partial) crystallization and structural relaxation of the amorphous state, we perform our measurements below room temperature i.e. between 140 K and 300 K. We derive temperature-dependent values for the inter-trap distance under illumination and in darkness by analyzing our data with a PF-based model for subthreshold transport. We relate this inter-trap distance to the electronic DoS by calculating the average distance between the various trap states in amorphous GST based on a DoS reported in literature.

## Results and Discussion

### Density-of-states for amorphous GST

In general, amorphous semiconductors can exhibit a wide variety of defect-states in their band-gap. As a result of disorder in bond-distances and angles amorphous materials feature bandtail-states^31^ which are characterized by an exponential decay of the defect-density *N*_*V*_ with energy *E* below the band-edge, *N*_*V*_ = *N*_*V*,0_ ⋅ exp(−*E*/*E*_*U*_). Therein, *E*_*U*_ is the decay constant on the order of 30 meV to 70 meV^32^. In addition to these tail-states, other structural defects can appear at specific energies in the band-gap. These are constituted by over- or under-coordinated atoms, e.g. Si in a-Si:H^33^,^34^ or Ge in GeTe^35^,^36^. These states are commonly modeled with Gaussian distributions, 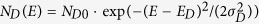, and are thus characterized by an energy-level *E*_*D*_, peak density *N*_*D*0_ and width *σ*_*D*_.

Amorphous GeTe and GST share key electronic features. Modulated photocurrent spectroscopy (MPC) experiments demonstrated the presence of tail-states as well as a shallow (*E*_*D*,*s*_ ~ 0.25 eV) and a deep defect (*E*_*D*,*d*_ ~ 0.4 eV) in both p-type semiconductors^23^,^37^,^38^. Moreover, the shallow defect could be described with the same attempt-to-escape frequency (a measure for its ability to capture holes from the valence-band) in both GeTe and GST^37^. For the valence-band tail, the characteristic energy and the attempt-to-escape frequency were also found to be the same in both materials^38^. In the case of GeTe, the characteristic energy of the conduction-band tail was inferred from photothermal-deflection spectroscopy which allowed for a more detailed modeling of the DoS^23^. In what follows, we exploit the similarity of key electronic features in the two materials and combine the experimental findings for the DoS of GST with additional evidence derived only for GeTe (e.g. the Urbach-energy *E*_*U*_ of the conduction band tail-state). An important difference between the materials is the absolute value of the MPC-DoS *NC*_*p*_/*μ*_0_ (*N* density, *C*_*p*_ capture-rate and *μ*_0_ the mobility of holes in the valence-band) of the shallow and deep defect (smaller in GST) and the photoconductivity (higher in GST)^38^,^39^. We account for this difference by assuming a lower DoS *N* for both shallow and deep defect. A more detailed account of all the parameters and their origin is given in the methods section.

Another important aspect for charge-transport in amorphous PCMs is the strong temperature dependence of the band-gap *E*_*G*_(*T*), which amounts to −79 meV absolute or −9% relative change from 150 K to 300 K in GST^37^,^40^,^41^, compared to −3% in a-Si:H^42^. Several studies have demonstrated that the observed temperature-dependence of the activation energy for conduction *E*_*A*_(*T*) can be modeled in a wide temperature range (200 K to 400 K) by assuming that the decrease of the bandgap with increasing temperature manifests directly in a proportionally temperature-dependent activation energy i.e. *E*_*A*_(*T*) = *b* ⋅ *E*_*G*_(*T*), where *b* < 0.5^24^,^41^,^43^,^44^,^45^.

How exactly the temperature dependence of *E*_*G*_(*T*) affects the defect-states in the DoS is subject of current research. In analogy to refs 37 and 44, we investigate here a situation in which the energetic distance between the valence-band edge *E*_*V*_(*T*) and the conduction-band edge *E*_*C*_(*T*) is given by the experimentally determined optical band-gap *E*_*G*_(*T*) and the energetic position *E*_*D*_(*T*) of both shallow and deep defect are proportionally scaled by *E*_*G*_(*T*), i.e. *E*_*D*_(*T*) = *c* ⋅ *E*_*G*_(*T*), where *c* is a constant for the respective defect and *E*_*D*_(*T*) its computed energetic distance to the valence-band edge. Conceivable alternative scenarios (temperature dependence of solely *E*_*V*_(*T*) or *E*_*C*_(*T*)) over- or underestimate the temperature-dependence of the activation for conduction and the inter-trap distance, see supplementary note S1.

### Steady-state occupation in the DoS for amorphous GST

**Figure 1:**
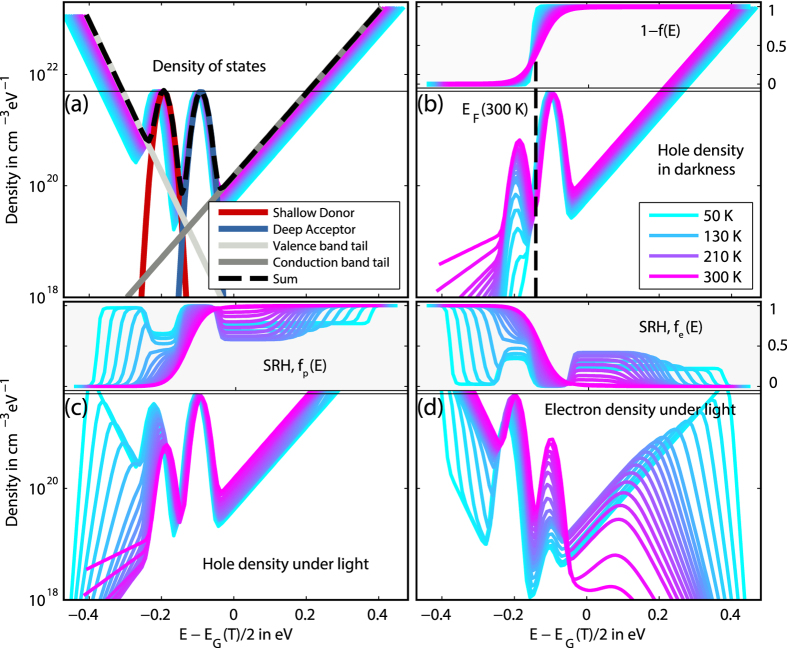
Density of states and its occupation. Top left panel, (**a**): Model DoS for GST at various temperatures indicated by colors from blue (50 K) to purple (300 K). To underline the temperature-dependence of the DoS, 18 different lines in steps of Δ*T* = 20 K between 50 K to 150 K, Δ*T* = 10 K between 160 K to 240 K as well as Δ*T* = 25 K between 250 K to 300 K are shown in total. The association between color and temperature is the same in all figures. The individual states (e.g. thick red, blue and grey curves) comprising the whole DoS (dashed curve) are shown at 300 K. The energy zero marks the middle of the bandgap in all panels. A straight horizontal line indicates the maximum defect density of the Gaussian states, *N*_*d*_ = 5 × 10^21^ 1/cm^3^eV. Top right panel, (**b**): Equilibrium density of unoccupied states for various temperatures. The Fermi energy *E*_*F*_ is indicated by the dashed line. The occupation function for holes 1 − *f*(*E*) is shown in the inset. Lower panels: density of unoccupied states (left panel, (**c**)) as well as density of occupied states both under illumination (right panel, (**d**)). The Shockley-Read Hall distribution function (calculated from the number of holes and electrons in the band and the capture coefficients) is shown in the respective inset to illustrate its influence on the occupation under illumination, as exemplified by the temperature-dependence of the occupation of the shallow defect.

We calculate the charge-carrier densities in darkness and under illumination for our specific DoS with the program DeOST^23^ under the constraints of charge-neutrality and equality of generation- and recombination-rate, cf.^46^. We assume that recombination is possible between states in the bands and defect-states but not among defect-states, an approach that leads to successful descriptions of transport under illumination in PCMs^23^,^37^,^44^. Moreover, we derive the occupation of the defects (Fig. 1) via the Shockley-Read Hall distribution function^46^ based on the density of charge-carriers in valence and conduction-band.

With the energy scale centered around the middle of the bandgap the strong p-type nature of the model system as well as the temperature-dependent shift of the energy levels and concurrent changes in occupation become visible. With regard to the temperature-dependence of the equilibrium occupation of holes (top right panel, (b) in Fig. 1), we observe that the density of holes in the shallow defect increases strongly with increasing temperature, whereas the density of holes in the deep defect does not vary much in the same temperature range. As the Fermi-energy *E*_*F*_ is pinned between the two Gaussian defects (see dashed line in top right panel and distribution function in the inset), the temperature-dependence of the density of holes in the shallow defect and its absolute number both can be observed for the electrons occupying the deep defect analogously (not shown). Above *E*_*F*_, the density of holes is mainly affected by the temperature-dependence of the bandgap, whereas there is a strong influence of the exponential energy- and temperature-dependence of the distribution function *f*(*E*) below *E*_*F*_. With increasing temperature *E*_*F*_ shifts toward midgap (cf. Fig. 1(b)) and, at the same time, closer to *E*_*V*_ as a result of relating the temperature-dependence of the bandgap *E*_*G*_(*T*) to the energy levels in the DoS in a proportional way.

Under illumination (bottom panels of Fig. 1), the occupation is governed by the Shockley-Read Hall occupation functions (top inset) for electrons *f*_SRH,*n*_ and holes *f*_SRH,*p*_, respectively. At low temperatures (cyan curves), charge-carriers that are generated by illumination are favorably distributed to defects with higher capture-rates e.g. holes to the valence band tail (*C*_*p*_ = 5 × 10^−10^ cm^3^/s) rather than to the shallow defect (*C*_*p*_ = 2.5 × 10^−11^ cm^3^/s), inset to Fig. 1(c). At higher temperatures (pink curves) thermal emission prevails and, consequently, the functional form of these distributions is very similar to the corresponding equilibrium distributions *f*(*E*) and 1 − *f*(*E*) with *f*(*E*) the Fermi-Dirac distribution. Regarding the occupation, we observe that the density of holes in the shallow defect decreases (cf. Fig. 1(c)) and the density of electrons in the deep defect increases (cf. Fig. 1(d)) with increasing temperature. The occupation of the tail states with electrons and holes is also found to be strongly temperature-dependent. At low temperatures, the occupation of these states is strongly affected by the temperature-dependent shift of the respective quasi-Fermi levels (cf. shift of maximum of cyan curves in lower panels).

For identifying which defect states are related to the intertrap distance, we find that only two scenarios are suitable: the occupation of the shallow defect with holes and the deep defect with electrons. All other scenarios yield inter-trap distances that are either too small and almost constant with temperature because the defects are fully occupied (as is the case for electrons in the shallow defect and holes in the deep defect) or too large and too strongly temperature-dependent because they are too far away from *E*_*F*_ (tail-states), cf. supplementary note S2. To quantify the temperature-dependence of the occupation more precisely, we calculate the inter-trap distance by integrating over the (un)occupied states of the two defects under consideration as 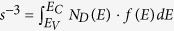, with the respective density of states *N*_*D*_(*E*) and distribution function *f*(*E*).

**Figure 2:**
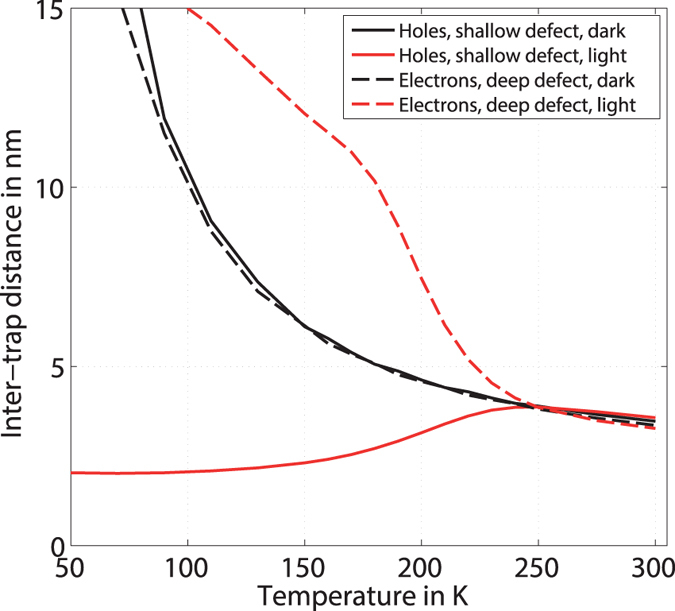
Inter-trap distance derived from occupation of the DoS. Temperature-dependence of the inter-trap distances *s* in darkness (black) and under illumination (red) for the shallow (*E*_*D*,*s*_ = 0.25 eV) and deep defect (*E*_*D*,*d*_ = 0.39 eV). The different line-styles denote the charge-carrier type and defect under consideration.

Comparing the inter-trap distance in darkness (*s*_*dark*_) and under illumination (*s*_*light*_), we find that *s*_*dark*_ and *s*_*light*_ differ significantly at low temperatures but become comparable at high temperatures, see black and red lines in Fig. 2. This implies that at room temperature the current under light (*I*_*light*_) is dominated by the dark current (*I*_*dark*_), whereas the difference between *I*_*dark*_ and *I*_*light*_ increases with decreasing temperature. Different experimental observations of the steady-state photoconductivity of PCMs^37^,^44^ (and our own below) confirm this expectation.

As expected from the temperature-dependent occupation (Fig. 1), *s*_*dark*_ increases with decreasing temperature. Furthermore, the distance between electrons in the deep defect is equal to the distance between holes in the shallow defect. Since the densities of the two defect-states are the same, this means that the Fermi-level is located between the two defects throughout the whole range of temperatures.

Under illumination, however, the calculated inter-trap distance *s*_*light*_ decreases with temperature for the holes in the shallow defect whereas it increases for the electrons in the deep defect. In both cases, an easy analytic description is not possible for *s*_*light*_ due to the strong shift of the quasi-Fermi levels with temperature, evidencing a nontrivial dependence of the distribution functions on temperature.

To summarize our modeling result, we expect the inter-trap distance under light *s*_*light*_ and in darkness *s*_*dark*_ to be similar at high temperatures. Within our model, decreasing temperature should lead to an increase of *s*_*dark*_ for both considered scenarios. Under illumination, the occupation of the shallow defect with holes should lead to a decrease of the inter-trap distance with decreasing temperatures, whereas the occupation of the deep defect with electrons should lead to an increase of the inter-trap distance with decreasing temperatures.

### Transport analysis

To extract the inter-trap distances from measured current-voltage characteristics (IV-curves), we employ a previously introduced transport model^24^,^45^. In this model multiple-trapping transport is combined with field-enhanced emission, building on works by Hill^11^ and Pillonet^22^. We refer to the methods section for a mathematical description. Here, we focus our discussion on the central conceptual points.

The model considers thermally-activated and field-assisted emission of a single charge-carrier trapped in a localized state in the bandgap. The potential localizing the charge-carrier is modified by the Coulombic potential of a neighboring defect. The distance *s* between these two defects affects the amount of overlap of the two potentials and thus the potential barrier the charge-carrier needs to overcome to be released from its trap.

The barrier for emission is given by *E*_*A*_ − *E*_*PF*_, i.e. the energy needed to reach the valence band reduced by barrier lowering due to both the applied electric field and a neighboring defect. Upon emission into the extended states, charge carriers move with band-mobility *μ*_0_ (details in the methods section) in direction of the electric field irrespective of the direction of emission from the trap state. The number of charge carriers *n*(*F*, *T*) activated over the potential barrier and the mobility *μ*_0_ are related via the conductivity as *σ* = *n*(*F*, *T*) ⋅ *q* ⋅ *μ*_0_. The current is computed via *I* = *σ* ⋅ *A* ⋅ *F*, with *A* the device cross-section.

The combination of these basic assumptions about the field-dependent emission and the subsequent transport process is able to reproduce the salient features of IV-curves of amorphous GeTe and GST in a wide range of temperatures (200 K to 400 K) in the meltquenched as well as in the as-deposited state^24^,^45^,^47^. It can even capture the effects of structural relaxation in melt-quenched devices^47^.

**Figure 3:**
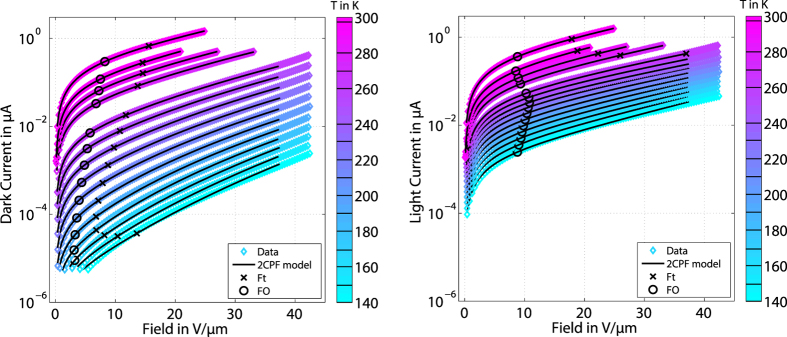
Field- and temperature-dependent device current in darkness and under illumination. Left: Dark current as a function of electric field (*F* = *V*/*l*, device length *l* = 2 μm) measured at various temperatures from 140 K to 300 K as indicated by solid lines in the colorbar. To prevent switching of the device a fixed current limit (~0.6 μA) was used at higher temperatures (except at 295 K which was the last measurement in this series). The fitted model is indicated by the straight black lines. Crosses and circles denote the transition field *F*_*O*_ from Ohmic to Poole, and *F*_*t*_ from Poole to Poole-Frenkel field-dependence. At the lowest temperatures and fields our model does not describe the data well. At extremely high fields (*F* ~ 40 V/μm), the field-dependence becomes stronger, cf. curve at 240 K. Right: Current under light. The different values of the transition fields indicate a marked difference between the inter-trap distances in darkness and under light. At high temperatures, the values become similar as the difference between *I*_*light*_ and *I*_*dark*_ vanishes.

Most importantly, the model can describe the three different regimes of field-dependence that have been observed in PCMs^24^,^45^,^48^,^49^,^50^: While the conductivity is constant at low fields (Ohmic regime), a Poole-Frenkel (PF) type behavior is observed at high fields (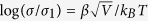). Between these two regimes, the conductivity exhibits an exponential field-dependence i.e. log(*σ*/*σ*_0_) = *eFs*/2*k*_*B*_*T* (Poole). The field of transition from Poole to Poole-Frenkel *F*_*t*_ = (*β*/*es*)^2^, with 
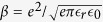
, depends on the inter-trap distance^21^. This transition field *F*_*t*_ can be marked in the fit to an experimentally recorded IV-curve serving as a graphical representation of the inter-trap distance (cf. Fig. 3). In a similar way, the field at which the transition from the Ohmic to the Poole regime occurs also depends on *s*, but additionally on temperature, *F*_*O*_ ∝ *k*_*B*_*T*/*es*, cf.^24^.

In the fitting approach of the present study we keep all parameters constant that are not related to the occupation of defects in the band-gap, i.e. they are set to be identical for both the dark current and the current under light. This includes device-related (geometry) and material-related parameters (dielectric constant 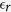, mobility *μ*_0_ and effective DoS at the valence band edge *K*_*PF*_). While the geometry of the devices is known, we infer the dielectric constant from previous measurements (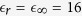 with negligible temperature-dependence^41^). With regard to *μ*_0_ and *K*_*PF*_ we note that, in absence of reported values for the charge-carrier mobility, the individual contribution of *μ*_0_ and *K*_*PF*_ cannot be disentangled because the two quantities enter the conductivity as factors (cf. transport model section). Thus, we infer a temperature-independent value for the product of the two quantities (*μ*_0_ ⋅ *K*_*PF*_ = 1 × 10^22 ^1/mVs) from previous measurements^24^. In consequence, our modeling approach features the two unconstrained parameters *E*_*A*_ and *s* for each IV-curve in darkness and under light.

Before we describe the results of such fits, we justify why transport should be limited by field-enhanced emission from trap states even when charge carriers are excited into the bands by photon absorption i.e. under illumination. To estimate for how long holes can travel after they are generated in the valence band by illumination, we recall that they are trapped into the valence-band tail with a trapping rate of *ν* = 2 × 10^12^ 1/s^23^. Assuming a band mobility of *μ*_0_ = 1 cm^2^/Vs, we compute the travel-distance *d* = *μ*_0_ ⋅ *F*/*ν* to be *d* = 1 nm at a field of *F* = 20 V/μm. Employing the smallest trapping-rate of the considered defects (the shallow defect, *v* = 1 × 10^10^ 1/s) yields an upper boundary for the traveling distance *d*_*s*_ ~ 200 nm. These larger distances are still considerably smaller than our device and, consequently, charge-carriers will be trapped repeatedly on their way from one electrode to the other. Hence, we expect that the model (field-enhanced emission, subsequent transport and re-trapping) can capture the transport under illumination, too.

### Experimental results

**Figure 4:**
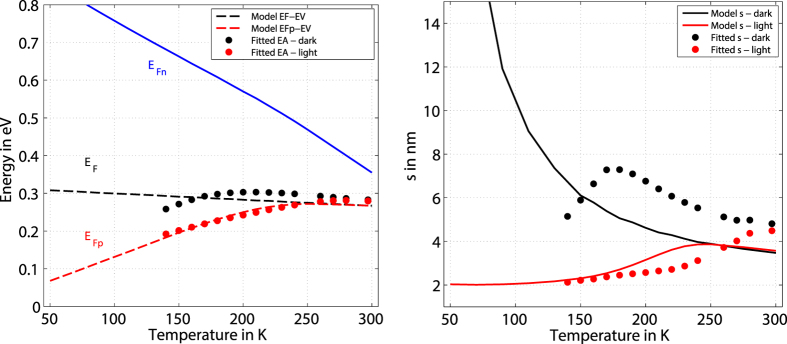
Comparison of measured and modeled activation energies and inter-trap distances. Left: Temperature dependent Fermi-energy *E*_*F*_ (black dashed line) and quasi-Fermi levels for electrons (blue) and holes (red) calculated from our model DoS. Activation energies extracted from the fit of the transport model to the data are indicated by black (darkness) and red (under illumination) circles. Right: Computed (lines) and fitted (circles) values for the inter-trap distances.

In line with the above reasoning, the model does yield a very good description of the measured current-voltage characteristics both in darkness and under illumination, cf. determined fit functions in Fig. 3. We observe that the transition fields *F*_*O*_ and *F*_*t*_ are higher under illumination than in darkness (cf. circles and crosses in Fig. 3). As mentioned above, this implies that the inter-trap distance is smaller under illumination than in darkness. To investigate the parameters derived from the two measurement series in more detail, we compare the fitted values for the activation energies *E*_*A*,*dark*_ and *E*_*A*,*light*_ with the values for *E*_*F*_ and the quasi-Fermi level for holes *E*_*Fp*_ and electrons *E*_*Fn*_ calculated for the DoS (left panel of Fig. 4).

In general, there is a good agreement between *E*_*A*,*dark*_ and *E*_*F*_ as well as between *E*_*A*,*light*_ and *E*_*Fp*_. We observe that *E*_*Fp*_ < *E*_*F*_ at all except the highest temperatures where *E*_*Fp*_ ~ *E*_*F*_. This implies that *I*_*light*_ ~ *I*_*dark*_ at 300 K as well as *I*_*light*_ > *I*_*dark*_ at lower temperatures, in line with the experimental observation (cf. Fig. 3).

The temperature-dependence observed in *E*_*A*,*light*_ and *E*_*Fp*_ is also in accordance with the defect occupation shown in Fig. 1: As *E*_*Fp*_ increases with increasing temperature the maximum of the occupied shallow defects shifts away from the valence band. Despite the good general agreement of fitted and modeled activation energies, there are slight discrepancies in both *E*_*A*,*dark*_ and *E*_*A*,*light*_ at high temperatures and more pronounced deviations at low temperatures in *E*_*A*,*dark*_. The decrease of *E*_*A*,*dark*_ below 200 K is in line with numerous previous observations of a decreasing activation energy of conduction that cannot be explained with the conduction model used^38^,^44^,^45^. One possible explanation could be a gradual transition towards hopping conduction accompanied by a decrease in activation energy, as has been proposed by Krebs *et al*. for GeTe^44^.

At high temperatures, our data suggest an activation energy which slightly decreases towards higher temperature. In our model, this decrease is reproduced by the temperature-dependence of the DoS, manifested in a strongly temperature-dependent optical band-gap *E*_*G*_(*T*). From previously published infrared spectroscopy measurements, we derive a change of *E*_*G*_(*T*) of ~ −50 meV in a range from 200 K to 300 K, which corresponds to roughly double of what we would determine from the activation energy. This corroborates our assumption that the temperature-dependence of *E*_*G*_(*T*) does neither propagate fully to *E*_*V*_(*T*) nor exclusively to *E*_*C*_(*T*) but rather affects all energy levels in general and those of the Gaussian defects in particular, cf. supplementary note S1.

Comparing the temperature-dependence of the extracted values for the inter-trap distance *s*_*light*_ and *s*_*dark*_ (right panel of Fig. 4) we observe that our general expectation from the calculation of occupied states regarding *s* is matched: a) the inter-trap distances *s*_*dark*_ and *s*_*light*_ are matched well within the accuracy we expect for our DoS (estimated from the variation of the DoS-spectroscopy data published so far^23^,^37^,^38^, cf. DoS modeling section) at high temperatures but differs more strongly at low-temperatures, b) *s*_*dark*_ increases with decreasing temperature (above 200 K) and c) *s*_*light*_ decreases with decreasing temperature.

Observations a) and b) demonstrate that the inter-trap distance depends on temperature and illumination. Thus, they address the first of the two fundamental questions we set out to answer, i.e. whether the inter-trap distance is a quantity related to the occupation of defects. In light of the above, we formulate our main claim: it is not the mere density of defect-states but rather their state of *occupation* that must be employed in transport-models for PCMs. With regard to the second question/objective (identifying a specific defect related to the inter-trap distance), observation c) might lead us to assume that the occupation of the shallow defect with holes should be identified as the traps defining the inter-trap distance (Fig. 2).

### Influence of charge-state and capture-coefficients

In the following, we want to address a subtle point related to both the charge-state of defects in our model and the values of the capture coefficients as reported in ref. 23. A peculiarity of our result is the observation that the states in the shallow defect that are occupied with a hole, i.e. unoccupied with an electron, seem to be related to the inter-trap distance. Since the shallow defect is a donor state in our model, it is more positively charged when occupied with a hole compared to when occupied with an electron.

If these positive charges were responsible for creating the overlapping Coulomb potentials that enter into our transport model, the charge carriers attracted by the potential wells would be electrons. Since amorphous GST is p-type^51^ and thus holes dominate the conductivity, it is unlikely that a positively charged donor is related to the inter-trap distance. Instead, the occupation of the deep acceptor would yield the necessary charge state i.e. more negatively charged when occupied with an electron.

As we have demonstrated, the temperature-dependence of the occupation of a deep acceptor can explain the measured temperature-dependence of *s*_*dark*_. The discrepancy between the observed temperature-dependence of *s*_*light*_ and that calculated for the deep acceptor (increase of *s*_*light*_ with decreasing temperature) could indicate that the parameter values affecting the steady-state occupation of that defect are inaccurate/incorrect. These could for example be the capture-rates of the deep acceptor.

The importance of the choice of the capture-rate for the Shockley-Read Hall distribution function for the deep defect *f*(*E*) becomes clear when we write it as *f* = 1/(1 + *p*/*rn*)^46^. Therein, *p* is the number of holes in the valence-band, *n* the number of electrons in the conduction-band and *r* = *C*_*n*_/*C*_*p*_ the ratio of the capture-rates of electrons and holes. The expression is valid for a given defect in between the two quasi-Fermi levels *E*_*F*,*p*_ and *E*_*F*,*n*_.

Thus, the distribution function is a constant in between *E*_*F*,*p*_ and *E*_*F*,*n*_. The value of *f* depends on *r* and the ratio *p*/*n*, and thus on temperature. Therefore, the deep defect could be occupied by significantly more electrons than we have calculated and we would have overestimated the inter-trap distance for the deep defect.

**Figure 5:**
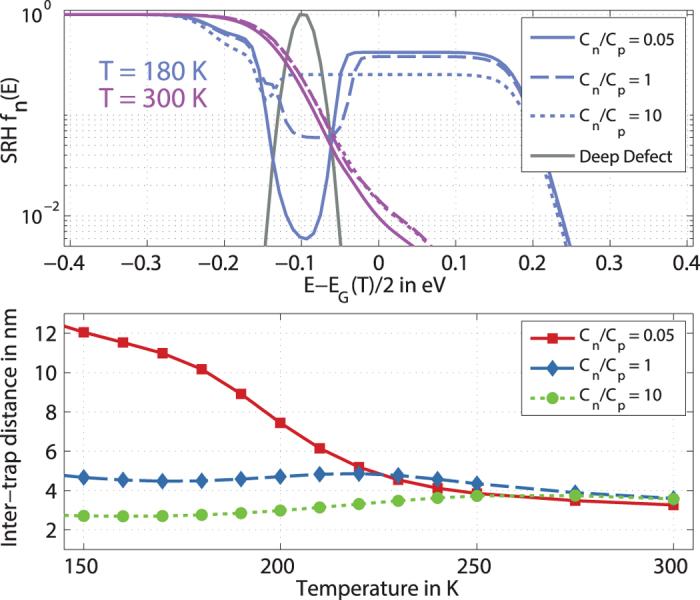
Impact of capture-coefficients on occupation and inter-trap distance. Top panel: Influence of the ratio of the capture-rates *C*_*n*_/*C*_*p*_ and temperature on the Shockley-Read Hall distribution function *f*(*E*) for the deep defect encoded in line-style and color, respectively. Lower panel: Temperature-dependence of the calculated inter-trap distance for the same ratios of *C*_*n*_/*C*_*p*_ as in the top panel.

To visualize these considerations, we show in Fig. 5 the occupation of electrons for three different ratios of *r* = *C*_*n*_/*C*_*p*_, *r*_1_ = 0.05, *r*_2_ = 1, *r*_3_ = 10, two different temperatures (*T* = 180 K and *T* = 300 K) and the corresponding inter-trap distance (lower panel).

While the variation of *r* does not strongly affect the occupation at high temperatures, it does so at lower temperature. Although the number of holes in the band (and thus the conductivity) does not vary much between the three cases (the density of holes in the band for case *r*_1_ is 2x higher than for *r*_3_), the calculated inter-trap distance varies significantly solely due to a variation in the capture-rates of the deep acceptor.

For *r* = 10, we observe that the inter-trap distance is in the right range of values and decreases with temperature, in agreement with experimental data. Apparently, the differences in the occupation (cf. top panel in Fig. 5) due to the variation of the capture coefficients induce pronounced differences in the absolute value and the temperature dependence of the inter-trap distance under light. This demonstrates that a more refined model of the capture-rates and more precise experimental data from DoS-spectroscopy are necessary to unambiguously relate the inter-trap distance in subthreshold conduction models to a specific defect.

### Implications

Two possible implications of our work on PCM research shall be mentioned in the following. As we have shown above, it is necessary and physically plausible to include a temperature-dependent inter-trap distance *s* in a model that describes temperature- and field-dependent conduction based on the PF-effect invoking such a distance *s*. Although we have focused on temperatures below 300 K, the argument can be extended to higher temperatures. From 200 K to 300 K we determined a relative change in s by a factor of 2 (cf. Fig. 4), whereas we estimate a relative decrease of 30% in a temperature range between 300 K and 400 K. Although the trend weakens, it still amounts to more net change than what is observed e.g. during resistance drift of the melt-quenched state^24^. It might thus become relevant when conduction is to be modeled in environments where changes of temperature occur. More importantly, the correct description of temperature-dependence of both the inter-trap distance specifically and the DoS in general are relevant when the combined influence of field and temperature on switching is described, as e.g. in ref. 20.

Our work also suggests a strategy for the design of new phase change materials aiming at a modified field-dependence of PRAM devices: When changing the inter-trap distance (defect density) via doping, dopants with energy levels in the vicinity of the Fermi-level can be expected to minimize the temperature-dependence of the distance between (un)occupied states, and hence the variation of the field-dependent conduction with temperature. Thus, dopants that stabilize the melt-quenched state against resistance drift are not necessarily good for PRAM performance if they introduce states far away from the Fermi-level leading to a stronger temperature-dependence of the inter-trap distance. In this regard, our present work provides a bridge between material science and engineering of devices: While the original^16^ and subsequently improved^24^ descriptions of the PF-effect as applied to PRAM incorporated the inter-trap distance merely as a fitting parameter, our work proposes a physical interpretation. The latter implies that studies of the electronic DoS and the constituent defect-states could yield valuable input to tailor device-behavior (i.e. the field-dependence) in a more deliberate way.

## Conclusion

In this study we have investigated the relation between the DoS of amorphous GST and the subthreshold current-voltage characteristics of microscopic GST-devices. By comparing the temperature-dependence of the low-field current with numerical results for temperature-dependent occupation we a) reiterated that the temperature-dependence of the DoS in PCMs can lead to a decrease of activation energy with increasing temperatures and b) pointed out that the experimental evidence for the DoS of GeTe and GST indicates that the Fermi-level - contrary to widespread interpretation e.g. refs 52 and 53 - is not located at *E*_*G*_(*T*)/2 but significantly closer to the valence band.

By manipulating the occupation of defect-states with both temperature and illumination we further demonstrated that it is not the distance between defect-states but rather the distance between *occupied* defect-states that must enter as relevant parameter in the description of temperature- and field-dependent electrical-transport models of GST. Our analysis further suggests that either the shallow- or the deep-defect (and not the band-tails) is related to the inter-trap distance. For GST, we have also underlined the role of the capture-rates which mandate further study.

The importance of accounting for the distance between occupied states can be generalized to other materials in which the field-dependent conductivity is modeled based on PF-emission with interacting defects. Thus, modeling the subthreshold conduction in amorphous carbon^54^ or the leakage current in high-k dielectrics^55^,^56^ may serve as examples in which a temperature-dependence of the inter-trap distance is to be expected.

## Methods

### Experimental

In the employed devices, the active material has the shape of a rectangular bar (line) bridging two metallic tungsten electrodes which were fabricated by using optical lithography on sapphire substrates^45^. The (measured) geometry of the active material in the employed cell was length *l* ~ 2 μm and width *w* ~ 22 μm. Thin films (*t* ~ 60 nm) of GST were deposited using DC-magnetron sputtering from stoichiometric targets. After the deposition process (*P* = 20 W in 5.3 mbar argon atmosphere), the GST-layer was capped *in-situ* with a thin ~10 nm layer of (ZnS)_80_:(SiO_2_)_20_ to prevent oxidation. All electrical measurements were performed in a Janis ST-500-2UHT cryogenic probing station (also described in refs 24 and 44) evacuated to pressures *p* ≤ 1 × 10^−4^ mbar. Prior to current-voltage measurements (using a Keithley 2612B SMU in constant voltage mode), temperature was allowed to stabilize for at least 10 minutes. The systematic deviation in temperature was measured to be less than 4 K at 100 K. Light was provided by a laser diode emitting light of wavelength *λ* = 830 nm, corresponding to ~1.49 eV. Employing an optical-feed through, light was coupled into a single-mode tapered optical fiber inside the vacuum chamber. Due to its large numerical aperture NA = 0.42 and distance to the sample *z* = 2 mm, an area with diameter of ~850 μm is illuminated on the surface of the sample. Thus, our devices are illuminated homogeneously. The relation between laser-diode current and optical power was calibrated. The employed flux was Φ = 5 × 10^18^ 1/cm^2^/s. Any change in flux resulting from expansion/contraction of the sample chuck after changes of temperature was accounted for according to an existing calibration. Current-voltage characteristics were measured once in darkness and once under illumination. No transient effects were observed in the dark- or photocurrent response on the time scale of the measurement (~1 min). Temperature control of the system was not affected as a result of illumination. In addition to the measurements shown above, the photocurrent was measured at various values of incident flux. At room temperature, the dependence of flux was approximately linear and is captured by our purely electronic model, see supplementary note S5. In addition, we did not observe any irreversible changes of the dark-conductivity after prolonged illumination that is known to occur for some chalcogenides^57^. Moreover, space-charge effects are negligible for our samples, as verified by measuring devices of varying length^45^.

### Formulation of the transport model

The main ingredients for the transport model are the field-enhanced emission process over the field-dependent activation barrier and the subsequent motion of charge carriers in states with a higher mobility. The potential employed for the calculation of the activation barrier is formulated as


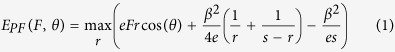


The angular dependence accounts for the notion that the charge carrier can also be emitted in some arbitrary direction (angle *θ*) to the field (upon emission it does move in direction of the field), cf.^12^,^58^. The term −*β*^2^/*es* (
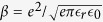
) is introduced so that the potential barrier height to be overcome corresponds to the experimentally recovered activation energy *E*_*A*_ in the low-field limit for arbitrary *s*, cf.^24^,^45^. For 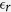, we use the high-frequency dielectric constant 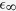 as derived from infrared-spectroscopy^41^, see supplementary note S3.

The field dependence of the charge-carrier density is attained by accounting for emission in all possible angles *θ* as


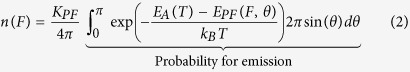


with *E*_*A*_(*T*) the temperature- and illumination-dependent activation energy at low fields. Thus, we use a Boltzmann-approximation to calculate the number of carriers. As result, the effective DoS at the valence-band edge *K*_*PF*_ appears in the above equation. Once a charge carrier is emitted, it can move with a possibly field-dependent mobility *μ*(*F*). For the melt-quenched phase, the concept (cf. discussion in ref. 24) of a field-dependent mobility (e.g. velocity saturation due to scattering with optical phonons) could slightly improve the description of the subthreshold transport at very high fields. Assuming a similar saturation velocity *v*_*sat*_ ~ 5 × 10^4^ m/s as in ref. 24 and a value of mobility *μ*_0_ = 1 cm^2^/Vs, the field at which velocity saturation would occur (*F*_*sat*_ = *v*_*sat*_/*μ*_0_ ~ 500 V/μm) is an order of magnitude higher than the threshold switching field in our devices (~60 V/μm). Consequently, we assume a field-independent mobility *μ*(*F*) = *μ*_0_, and hence a field-independent factor *K*_*PF*_ ⋅ *μ*_0_. Moreover, this factor should also be independent of temperature since a possible temperature-dependence of the effective DoS at the band-edge (*K*_*PF*_ = *k*_*B*_*TN*_*V*_, *N*_*V*_ DoS at the valence band edge) should be compensated by a temperature-dependence of the low-field mobility (*μ*_0_ ∝ *eh*/*k*_*B*_Tm^*^ 59), thus yielding a temperature-independent product *K*_*PF*_ ⋅ *μ*_0_. In fact, the value employed to describe transport in melt-quenched doped-GST^24^ also matches our data well, *K*_*PF*_ ⋅ *μ*_0_ = 1 × 10^22^ 1/mVs. We attempt further differentiation between the contribution of the effective DoS at the band-edge and the charge-carrier mobility in supplementary note S4. Finally, the conductivity is written as *σ*(*F*, *T*) = *n*(*F*, *T*)/*K*_*PF*_ ⋅ *e* ⋅ (*K*_*PF*_ ⋅ *μ*_0_) and the current related to the field via Ohm’s law as *I*(*F*, *T*) = *σ*(*F*, *T*) ⋅ *F* ⋅ *A*, with the cross section of the device *A* = 60 nm ⋅ 22 μm.

In summary, this leaves two free parameters that are affected by changes of occupation and hence temperature and illumination. These are the barrier for activation into states with higher mobility *E*_*A*_(*T*) and the inter-trap distance *s*(*T*) which are allowed to vary in the fit of the model to the data.

### DoS model and computation

According to refs 23 and 37, both GeTe and GST comprise an identical position of the shallow defect (*E*_*D*,*s*_ − *E*_*V*_ ~ 0.25 eV cf. refs 23 and 37) as well as the same attempt-to-escape frequency *ν*_*p*_ = *C*_*p*_ ⋅ *N*_*V*,eff_ = 1 × 10^10^ 1/s. Here, *C*_*p*_ is the capture-rate and *N*_*V*,eff_ the effective DoS at the valence band edge, i.e. *K*_*PF*_ in the transport model, see above. As also the absolute values of the MPC-DoS, the attempt-to-escape frequency for the valence band tail (VBT), *ν*_*VBT*_ = *C*_*p*,VBT_ ⋅ *N*_*V*,eff_ = 1 × 10^12^ 1/s, and the Urbach-energy *E*_*U*_ = *k*_*B*_ ⋅ *T*_VBT_ ~ 32 meV were found to be similar in both materials, we assume for GST *N*_*V*,eff_ = 3.9 × 10^21^ 1/cm^3^, i.e. as in GeTe. Moreover, both materials show similar values of the activation energy for conduction (0.30 eV to 0.35 eV) and thermopower (0.8 mV/K to 0.95 mV/K at 300 K, see ref. 51) which indicates a similar position of the Fermi-level *E*_*F*_. Assuming a heat of transport constant *A* = 1, *E*_*F*_ can be estimated as ~0.27 eV at 220 K and ~0.23 eV at 350 K, from the thermopower data in ref. 51.

The key difference between our DoS model for GST and that for GeTe^23^ is that we assume a lower (4x) defect-density of both shallow and deep defect for GST, i.e. *N*_*D*,*s*_ = *N*_*D*,*d*_ = 5 × 10^21^ 1/cm^3^/eV, accommodating for the experimental observation of a lower value of the MPC-DoS of these defects and a higher photoconductivity in GST than in GeTe^38^,^39^. We also note that the absolute values (and hence the relative difference) of the reported MPC-DoS of these defects in GST and GeTe varies considerably (from a factor of 2x to 80x^23^,^38^,^39^), possibly due to aging effects and/or differences in preparation conditions of the amorphous state). The modeling result of ref. 23 uses the highest reported values for the MPC-DoS and, consequently, the DoS of the Gaussian-defects we use here could be over- but probably not underestimated. Based on the ratio of the two highest MPC-DoS reported for the shallow defect in GeTe (*NC*/*μ* = 8 × 10^10^ V/cm^2^eV) and GST (*NC*/*μ* = 5 × 10^9^ V/cm^2^eV), we infer that an overestimation of the density by 4x and an underestimation of the inter-trap distance by 4^1/3^ ~ 1.5 could be realistic. This is in line with the underestimation of the inter-trap distance, cf. Fig. 4.

With regard to the deep defect, we confine our model to the experimentally observed single deep-acceptor distribution at *E*_*D*,*d*_ = 0.39 eV, instead of 2 acceptor distributions at slightly different energies, obtained as a modeling result in ref. 23. The capture-rates for electrons *C*_*n*_ = 5 × 10^−11^ cm^3^/s and holes *C*_*p*_ = 2.5 × 10^−12^ cm^3^/s are assumed as in ref. 23. A list of important parameters can be found in Table 1 - others are assumed as by Longeaud^23^. The charge-states of the (monovalent) defects in our model are either acceptor i.e. negatively charged when occupied with an electron (deep defect, conduction band tail) or donor i.e. positively charged when occupied with a hole (shallow defect, VBT).

According to the framework developed by Simmons and Taylor^46^, we calculate the steady-state distribution by enforcing charge-neutrality as well as equality of generation *G* and recombination rate *R*. To this end, we use the freely available software DeOST^60^. While we take *G* simply as the product of the absorption coefficient *α* (for GeTe at ~1.5 eV) and incident flux Φ, *G* = *α* ⋅ Φ, *R* is a function of the occupation of the states. When both conditions (*G* = *R* and charge-neutrality) are met, the conductivity under light is calculated from the number of holes in the valence band. The number of electrons in the conduction band is negligibly small for our DoS in the studied temperature range.

To calculate the density of occupied states as shown in the lower panels of Fig. 1, *N*_occ_(*E*) = *N*(*E*) ⋅ *f*_*SRH*_(*E*), we calculate the distribution function of each individual defect from its respective Shockley-Read Hall distribution function *f*_SRH_(*E*), as suggested by Simmons and Taylor^46^.

## Additional Information

**How to cite this article**: Kaes, M. and Salinga, M. Impact of defect occupation on conduction in amorphous Ge_2_Sb_2_Te_5_. *Sci. Rep.* **6**, 31699; doi: 10.1038/srep31699 (2016).

## Supplementary Material

Supplementary Information

## Figures and Tables

**Table 1:** Parameter values employed in the DoS-model.

DoS model	
Densities	*N*_*D*,*s*_ = *N*_*D*,*d*_ = 5 × 10^21^ cm^−3^eV^−1^
	*N*_*c*_(300 K) = *N*_*v*_(300 K) = 3.9 × 10^21^ cm^−3^
Capture rates	*C*_*p*,*s*_ = 2.5 × 10^−12^ cm^3^s^−1^, *C*_*n*,*s*_ = 5 × 10^−11^ cm^3^s^−1^
	*C*_*p*,*d*_ = 3 × 10^−11^ cm^3^s^−1^, *C*_*n*,*d*_ = 1.5 × 10^−12^ cm^3^s^−1^
Energies	*E*_*D*,*d*_(0 K) = 0.39 eV, *E*_*D*,*s*_(0 K) = 0.25 eV
	*E*_*D*_(*T*)/*E*_*G*_(*T*) = *E*_*D*_(0 K)/*E*_*G*_(0 K), other cases, see suppl.
	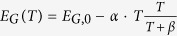 , *E*_*G*,0_ = 0.953 eV, *α* = 0.555 meVK^−1^, *β* = 65 K

The parametrization of the optical band-gap for GST is taken from ref. 41. All other DoS-parameters were taken from ref. 23.
