# Supplementary material for: Impact of defect occupation on conduction in amorphous Ge2Sb2Te5
Source: Sci Rep. 2016 Aug 16;6:31699. doi: 10.1038/srep31699 (PMC4985640; doi:10.1038/srep31699)
Supplement: Supplementary Information [file srep31699-s1.pdf]

Impact of defect occupation on conduction in amorphous $Ge_2Sb_2Te_5$ - Supplement

Matthias Kaes* and Martin Salinga†
I.Physikalisches Institut (IA), RWTH Aachen University, Sommerfeldstrasse 14, 52074 Aachen - Germany
(Dated: July 7, 2016)

S1 - INFLUENCE OF THE TEMPERATURE-DEPENDENCE OF THE DOS ON THE TEMPERATURE-DEPENDENCE OF THE ACTIVATION ENERGY

To demonstrate how our choice of accounting for the temperature-dependence of the DoS affects the temperature-dependence of the activation-energy for conduction and the inter-trap distance, we propagate the experimentally determined (cf. ref [1]) temperature-dependence of $E_G(T) = E_{G,0} - \alpha T^2/(T+\beta)$, a) to a temperature-dependence of the conduction-band edge i.e. $E_C(T) = E_G(T)$, b) to a temperature-dependence of the valence-band $\Delta E_V(T) = \Delta E_G(T)$ and c) to a temperature-dependence of the Gaussian defect states $E_D(T)/E_G(T) = E_D(0\,\mathrm{K})/E_G(0\,\mathrm{K}) = \mathrm{const}$ as well as the distance between the band-edges $E_C(T) - E_V(T) = E_G(T)$ (i.e. as in the main text). For each case, we compute the temperature-dependence of the unoccupied states in darkness (upper panels of figure 1) as well as the Fermi-levels in darkness and under illumination (middle panels of figure 1) and the temperature-dependence of the calculated inter-trap distance for the holes in the shallow defect and the electrons in the deep defect (lower panels of figure 1). In absence of experimental evidence to the contrary, we assume the band-tails to be temperature-independent. For simplicity, we do not change the energy-dependence of the tail states in the calculation of case c). Thus, the density of these states is temperature-dependent (cf. figure 1) because they are attached to the respective bands, $E_C(T)$ and $E_V(T)$.

Comparing the three different scenarios, we observe a pronounced shift of the position of the maxima of the occupied states relative to midgap in cases a) (E_C temperature-dependent) and b) (E_V temperature-dependent). While this shift is accompanied by a strong temperature-dependence of E_F for b), E_F is nearly independent of temperature for a) (cf. middle panel of figure 1) - as expected for our p-type model. For case c), the temperature-dependence of the occupation is less pronounced since the temperature-dependence is not propagated fully to the valence- or conduction-band. In line with this, the temperature-dependence of the Fermi-level is stronger than in a) but weaker compared to b). In a range from 200 K to 300 K, a decrease of $\Delta E \approx -14\,\mathrm{meV}$ is observed for c) compared to $\Delta E \approx -31\,\mathrm{meV}$ for b). Thermopower measurements indicate a decrease of $\sim 15\,\mathrm{meV}$ in the same temperature-range (estimated from thermopower data in [2] with a heat-of-transport constant A=1). In consequence, propagating the temperature-dependence of the DoS to E_C underestimates the temperature-dependence of the activation-energy. In contrast, propagating the temperature-dependence of the DoS solely to E_V overestimates the temperature-dependence of the activation-energy. Instead, scaling the defect-levels proportionally to the optical band-gap yields a good description of the temperature-dependence of the activation energy that is frequently observed in PCMs [2–5] and is also in line with DoS-spectroscopy data [6].

With regard to the temperature-dependence of the inter-trap distance, we observe that in all three scenarios the temperature-dependence of s_{light} is opposite for the two defects (cf. lower panels of figure 1). Thus, our conclusion that the occupation of the shallow defect with holes should be associated with the inter-trap distance s_{light} is unchanged by taking into account the temperature-dependence of the DoS in a different manner. As motivated in the main text, only the parameters associated with the steady-state occupation of the deep defect with electrons, e.g. the capture cross-sections, could reverse this trend.

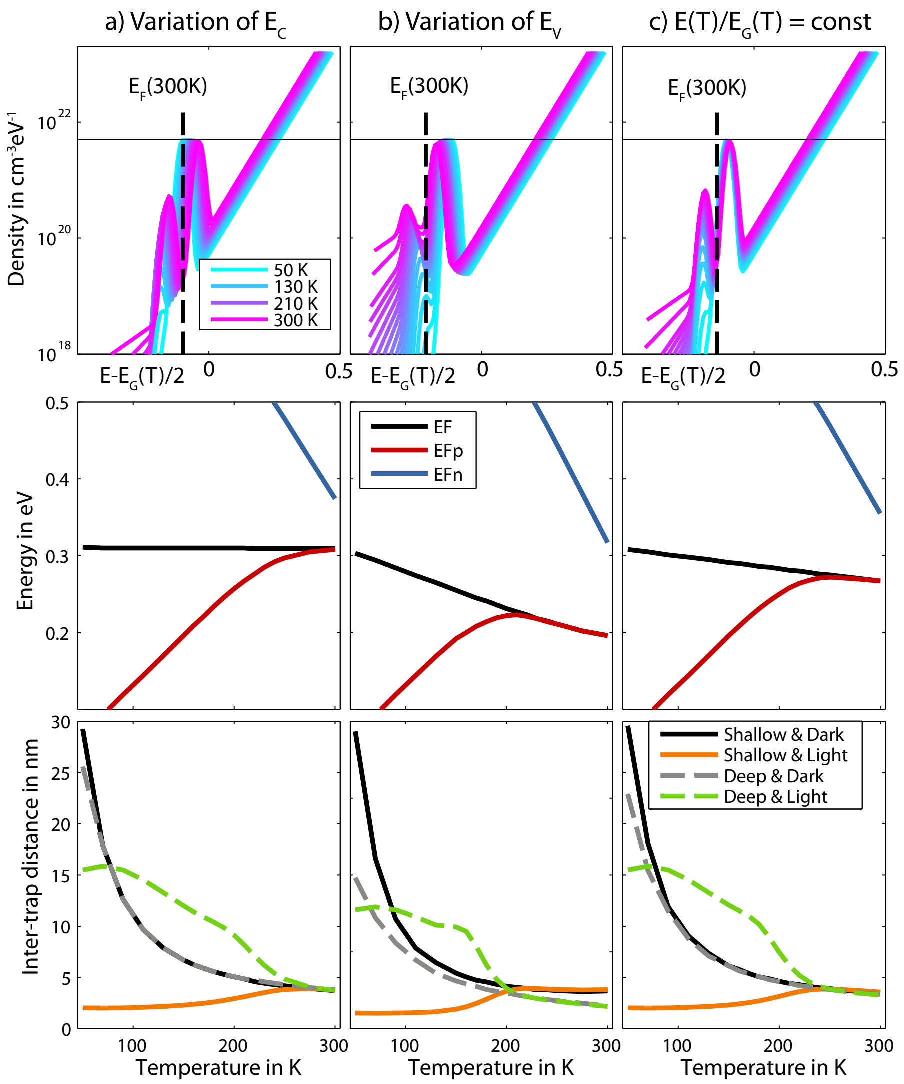

FIG. 1. Occupation, Fermi-energy and inter-trap distance in different scenarios of a temperature-dependent DoS: The three columns show the cases a) Temperature-dependent conduction band, b) Temperature-dependent valence band and c) Temperature-dependent Gaussian defect states with the position of the shallow defect $E_{D,s}(0\,\mathrm{K}) = 0.25\,\mathrm{eV}$ and the deep-defect $E_{D,d}(0\,\mathrm{K}) = 0.39\,\mathrm{eV}$. Upper panels: Occupation of holes for various temperatures with energy scale centered around midgap. Middle panels: Temperature-dependent Fermi level E_F and quasi-Fermi levels for electrons $E_{F,n}$ and holes $E_{F,p}$. Lower panels: Temperature-dependent inter-trap distances computed for the holes in the shallow defect (solid lines) and the electrons in the deep defect (dashed lines).

S2 - TEMPERATURE DEPENDENCE OF THE OCCUPATION OF THE TAIL STATES

To underline that the occupation of the tail states is not related to the inter-trap distance, we show calculated values for s_{dark} and s_{light} in figure 2. The values extracted from measurements are shown as circles. For comparison, the inter-trap distance related to the unoccupied states in the shallow defect is also shown (dotted line). Calculating the inter-trap distance related to the number of holes in the conduction band tail (dashed dotted line) leads to very low values $s_{light} < 1\,\mathrm{nm}$. Since the maximum of the product $f(E) \cdot N(E)$ of occupation function and DoS for these states is far above E_F irrespective of temperature and illumination (cf. figure 1 in the main text), the number of unoccupied states is very high and does not change much with temperature. The same argument holds for the number of electrons in the VBT. Thus, these states cannot be associated with the inter-trap distance extracted from experiment.

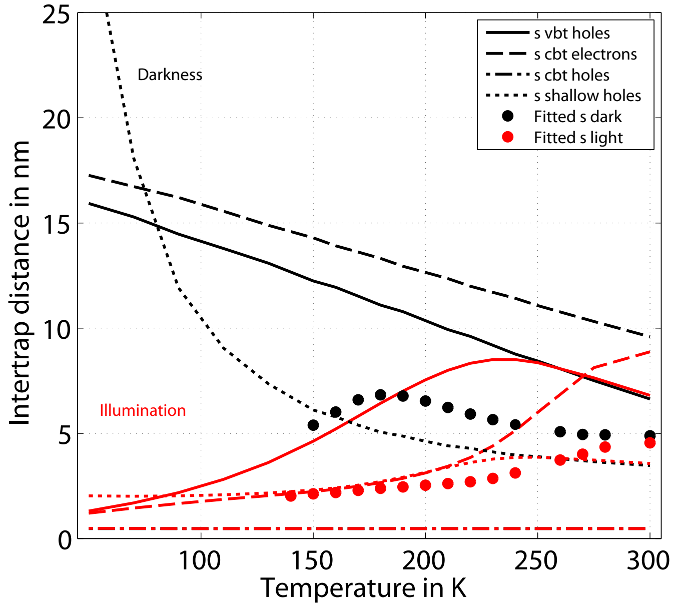

FIG. 2. Inter-trap distance associated with the tail-states:
Temperature-dependent inter-trap distance for various defect states (lines) in comparison to the values derived from experiment (circles) under illumination (red) and in darkness (black). The different computed scenarios are: inter-trap distance for holes in the VBT, electrons in the CBT, holes in the CBT. The inter-trap distance associated with holes in the shallow defect is shown for comparison.

Regarding the number of unoccupied states in the VBT, we observe a steady, almost linear increase of s_{dark} with decreasing temperature and rather large values $s \approx 11\,\mathrm{nm}$ at $200\,\mathrm{K}$, $s \approx 7\,\mathrm{nm}$ at $300\,\mathrm{K}$. The steady increase of s with respect to temperature is in contrast to the values determined from experiment, where we observe a flattening of the temperature dependence of s_{dark} at high temperatures. The difference in the characteristic shape is related to the difference in the energy dependence of the considered states, i.e. exponential in case of the tail state and Gaussian in case of the shallow defect.

With regard to s_{light}, we note that one striking difference between the occupation of the tail states and the shallow defect is that s_{light} becomes almost constant at low temperature. This is related to the quasi-Fermi level for holes which shifts over the shallow defect with increasing temperature, the defect being unoccupied at low temperatures and fully occupied at high temperatures. In contrast to that, the tail states cannot become fully (un)occupied because the quasi-Fermi level cannot be inside the valence or conduction bands. Thus, the inter-trap distance associated with these states is always temperature dependent.

In summary, the main features that speak against associating the inter-trap distance extracted from experiment with the tail states are 1) the lower overall density of (un)occupied tail states at high temperatures (in relation to the higher density of the shallow and deep defect in the relevant range of energies) and 2) the characteristic, almost linear temperature dependence of the calculated s_{dark} which cannot describe the data well. In contrast, the temperature-dependence of the experimental values of s_{dark} can be described much better when associating the inter-trap distance with the shallow or deep defect, i.e. defects which are confined to a narrow range of energies.

S3 - MOTIVATION OF THE CHOICE OF ϵ_∞

Conventionally, the high-frequency dielectric constant ϵ_∞ is used when describing PF-emission. This is related to the notion that the emission process of the charge-carrier from a bound-state is so fast that the remaining charge can be screened only by the electronic part of the dielectric response. For amorphous PCMs, it is important to verify whether this is the right interpretation as the difference between the high-frequency dielectric constant ϵ_∞ and the static dielectric constant ϵ_{st} is considerable. In the case of GeTe, the values are $\epsilon_\infty \sim 11.5$ [1] and $\epsilon_{st} \sim 25$ [7], both at $\sim 100\,\mathrm{K}$. For GST, measurements of ϵ_{st} are not available whereas an average value of $\epsilon_\infty \sim 16$ can be extracted from the measurements shown in ref. [1].

To demonstrate that this value also describes our data best, we recall that the employed conduction model predicts a high-field behavior of $\sigma(F,T) = \sigma_0(T) \cdot \exp(\beta\sqrt{F}/k_BT)$. As ϵ_r enters only in $\beta = e^2/\sqrt{e\pi\epsilon_r\epsilon_0}$, we test our model with various input values for ϵ_r, $\epsilon_1 = 16$, $\epsilon_2 = 24$ and $\epsilon_3 = 32$ and check the resulting match between model and data. We quantify this match by defining an error function $\chi(T,V) = \ln(I_{data}(T,V)) - \ln(I_{model}(T,V))$.

Figure 3 shows the recorded IV-curves at various temperatures as well as the fitted model functions for the considered values of ϵ_1 in figure 3a, ϵ_2 in figure 3b, ϵ_3 in figure 3c and the according net error $\sum_V |\chi(T,V)|$ in figure 3d. As expected, changing the value of ϵ_r influences the model function especially at high voltages. We observe that the mismatch between model and data becomes smaller when ϵ_r approaches the value of ϵ_∞ (see transition from figure 3c to figure 3b to figure 3a). The net error as defined above quantifies and corroborates this observation. In the temperature-range in which we expect our model to work well (above $\sim 180\,\mathrm{K}$) it becomes smallest when $\epsilon_r \sim \epsilon_\infty$.

In addition, forcing a high value of ϵ_r leads to a marked relative shift of the transition field F_t (x in figure 3) between the Poole- and the PF-regime, which decreases when increasing ϵ_r. The fit of the model to the data cannot absorb these changes because it would have to decrease the inter-trap distance to keep F_t constant, $F_t \propto 1/\epsilon_r s$ i.e. by a factor of 2 for $\epsilon_3 = 32$ compared to $\epsilon_1 = 16$. Increasing the value of ϵ_r, however, also leads to an underestimation of the current at high voltages and thus the fit accommodates for this underestimation by increasing the inter-trap distance, thereby increasing the current at intermediate voltages. As a result, the transition field F_t is reduced even further. Moreover, the increase of ϵ_r can also lead to a situation where the transition field from the Ohmic to the Poole regime ($F_O \propto k_BT/s$) is larger than F_t, cf. figure 3c. This inadequacy of the model can be taken as further indication that it is important to choose the "correct" value for ϵ_r, i.e. $\epsilon_r \sim \epsilon_\infty$ as demonstrated above.

S4 - IMPACT OF THE EFFECTIVE DOS AT THE BAND-EDGE AND ACTIVATION-ENERGY FOR CONDUCTION ON THE CHARGE-CARRIER MOBILITY

In absence of measurements for the charge-carrier mobility in amorphous phase-change materials, disentangling the contribution of mobility μ_0 and effective DoS at the valence-band edge K_{PF} to the conductivity is difficult. This is because both quantities enter the field- and temperature-dependent conductivity as factors $\sigma(F,T) = \mu_0(T)K_{PF}(T) \cdot \exp(-E_A(T,F))$. While it is reasonable to assume that the product of both is independent of temperature (cf. transport model section of main text), the absolute value is an adjustable parameter in our model, $\mu_0 \cdot K_{PF} = 1 \times 10^{22}\,\mathrm{m/Vs}$.

To determine the charge-carrier mobility, we match the conductivity in darkness σ_D and under illumination σ_L at low fields to the number of holes p_D and p_L (electrons are negligible) derived from our DoS-model. This yields a value of mobility of $\mu_0 = 0.04\,\mathrm{cm^2/Vs}$, which is rather low. Several estimations exist for the extended-states mobility in amorphous semiconductors, ranging from $1\,\mathrm{cm^2/Vs}$ to $10\,\mathrm{cm^2/Vs}$, [8, 9]. In what follows, we point out that a very plausible explanation for the obtained value could be a) an underestimation of the activation-energy and, more importantly, b) an overestimation of the effective DoS at the band-edge.

Expressing the conductivity as $\sigma_{D,L} = p_{D,L} \cdot e \cdot \mu_0$, we see that an overestimation of $p_{D,L}$ can easily lead to an underestimation of μ_0 to match a specific conductivity value. Writing the hole-density as $p_{D,L} = N_V \exp(-E_{A,D,L}/k_BT)$ (effective DoS at the valence-band edge N_V) implies that an overestimation of $p_{D,L}$ could result from an overestimation N_V or an underestimation of $E_{A,D,L}$. Indeed, we have observed a slight mismatch between modeled and fitted activation energies in figure 4 of the main text, $\sim 20\,\mathrm{meV}$. At room temperature, this can account for a factor of $\exp\left(\frac{20\,\mathrm{meV}\cdot q}{k_B \cdot 300\,\mathrm{K}}\right) \sim 2$.

However, the effective DoS at the valence-band edge we haven taken from ref. [10] can have an even larger impact. In the method that was employed in ref. [10] for extracting N_V, the inferred activation energy for conduction plays a crucial role. Following the notation of ref [10], this is because the product $\mu_p N_V$ in $\sigma = \mu_p N_V \cdot \exp((E_V(T) - E_F(T))/k_BT)$ was estimated from the 'apparent-activation energy' $E_{\mathrm{app}} = \partial \ln\sigma/\partial(k_BT)^{-1}$. Subsequently, E_{app} was

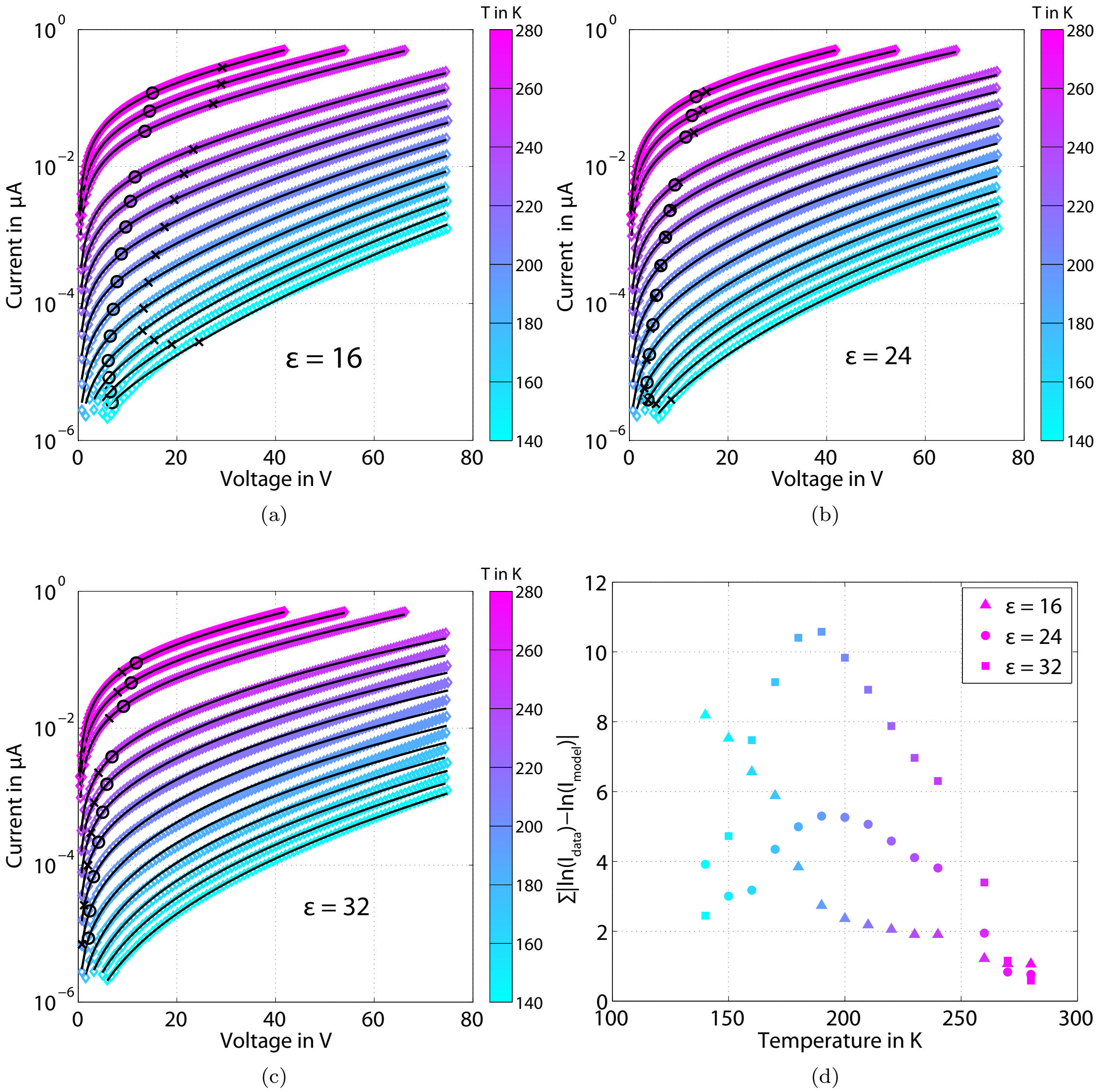

FIG. 3. Impact of varying the value of ϵ_r on the match between data and model:
Data at various temperatures as shown in the main text, only as function of the applied voltage a-c) Match between data and model for $\epsilon_1 = 16$, $\epsilon_2 = 24$, $\epsilon_3 = 32$ d) Fit error $\sum_V |\chi(T,V)|$ at constant temperature. Since the absolute difference between the data and model is highest at high voltages, the fit error as defined in the text is a good measure for the impact of changing the value of ϵ_r. The difference in absolute value of the error function arise because the absolute difference between model and data is temperature-dependent. As introduced in the main text, the transition fields (here voltages) between the Ohmic and the Poole, and the Poole and PF-regime are marked with circles and crosses, respectively.

taken to correspond to the actual activation energy $E_V(T) - E_F(T)$. This neglects a temperature-dependence of the DoS and can lead to an overestimation of E_A on the scale of $\sim 50\,\text{meV}$. At room temperature, this would lead to an overestimation of $\mu_p N_V$ by a factor of $\sim \exp(50\,\text{meV} \cdot q/k_B \cdot 300\,\text{K}) \sim 7$. In combination with the attempt-to-escape frequency $\nu = N_V \cdot C_p$, the value of $\mu_p N_V$ is further used to determine the ratio $\nu/\mu_p N_V = C_p/\mu_p$. This ratio is then employed to transform the MPC-DoS $N_V \cdot C_p/\mu_p$ into a 'real'-DoS, see [10]. Since ν and $N_V \cdot C_p/\mu_p$ are values that are constrained by the experiment, an overestimation of $\mu_p N_V$ thus leads to an overestimation of N_V.

These considerations underline that independent measurements of the low-field mobility of PCMs are clearly desirable. Within our present analysis however, the implications are of minor importance with regard to the temperature-dependence of the inter-trap distance. To demonstrate this, we separate the contributions to our conductivity model into terms that are field- and temperature-dependent ($E_{PF}(F,T,\theta)$) and terms that are only temperature-dependent ($\sigma_0 = \mu(T)K_{PF}(T)$, $E_A(T)$). Thus, we may write

$$\sigma(F,T) \propto \underbrace{\sigma_0 \cdot \exp\left(\frac{-E_A(T)}{k_B T}\right)}_{\text{T-dependent}} \cdot \underbrace{\int_0^{\pi} \exp\left(\frac{E_{PF}(F,T,\theta)}{k_B T}\right) \sin(\theta) d\theta}_{\text{T- and F-dependent}} \tag{1}$$

Since the contributions that are only temperature-dependent are captured with a single fit parameter for the current-voltage characteristics at each temperature ($E_A(T)$), the temperature-dependence of the field-dependent part $E_{PF}(F,T,\theta)$ can be determined independently.

The parameters that could affect the temperature-dependence of $E_{PF}(F,T,\theta)$ are the inter-trap distance $s(T)$ and the dielectric constant ϵ. As demonstrated in S3, the high-frequency dielectric constant ϵ_∞ can best describe our data. Our recent results from FTIR-spectroscopy (cf. ref. [1]) show that the temperature-dependence of the dielectric constant is very small in our range of temperatures ($\leq 4\%$ relative change from 150 K to 300 K) and thus we can neglect it here. In consequence, the only parameter that could capture a temperature-dependence of the field-dependent barrier-lowering E_{PF} is the inter-trap distance. Thus, our main argument (occupation of defects leads to a temperature-dependence of the inter-trap distance) is unaffected by an overestimation of the effective DoS at the valence-band edge. In fact, a smaller value of the DoS might even lead to a larger value of the inter-trap distances computed from the DoS model and hence further improve the match between computed and fitted inter-trap distances. To properly discuss the data available in literature we have refrained from adjusting the density at the band-edges. Thus, the above discussion may serve to highlight the need to account for temperature-dependent activation energies properly as well as to point out an open question in PCM research: the charge-carrier mobility.

S5 - IMPACT OF ILLUMINATION ON SAMPLE TEMPERATURE

So far, we have not addressed the impact that illumination with light has on the temperature of the sample. Our modelling approach does not account for such an influence and ascribes the change of current under illumination to a purely electronic origin: changes in the occupation of defect states. The changes depend both on temperature as well as on the generation rate of charge-carriers in the band, and hence the light flux. In fact, our model predicts an almost linear variation of the photocurrent I_{Ph} with flux. Expressing the dependence on flux Φ as a generic power-law $I_{Ph} = I_0 \cdot (\Phi/\Phi_0)^\nu$, we obtain an exponent of $\nu = 0.99$ at 300 K ($\nu = 0.9$ at 250 K) from our modeling approach detailed in the main text.

To assess whether we recover this exponent in the experiment, we measured the current-voltage characteristics at 300 K also for various light fluxes in the range from $1.5 \times 10^{18}\,1/\text{cm}^2\text{s}$ to $5 \times 10^{18}\,1/\text{cm}^2\text{s}$ (cf. figure 4). These are slightly lower compared to the experiment shown in the main text where $\Phi = 5 \times 10^{18}\,1/\text{cm}^2\text{s}$ was used. As expected, a decrease in flux leads to a decrease in both the current under illumination (figure 4a) as well as the photocurrent (4b). Moreover, we observe that the flux-dependence follows the expected power-law with an exponent $\nu \sim 0.9$ that is close to the predicted value of $\nu = 0.99$. This match between experiment and electronic model leads us to conclude that the intensity of the incident light is sufficiently low to neglect thermal influences that could occur a result of illumination.

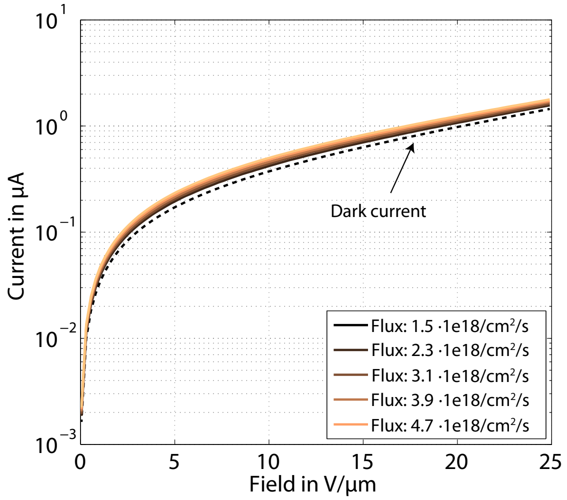

(a)

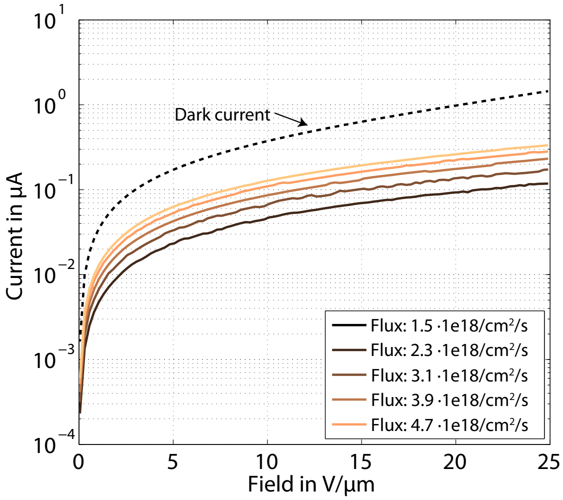

(b)

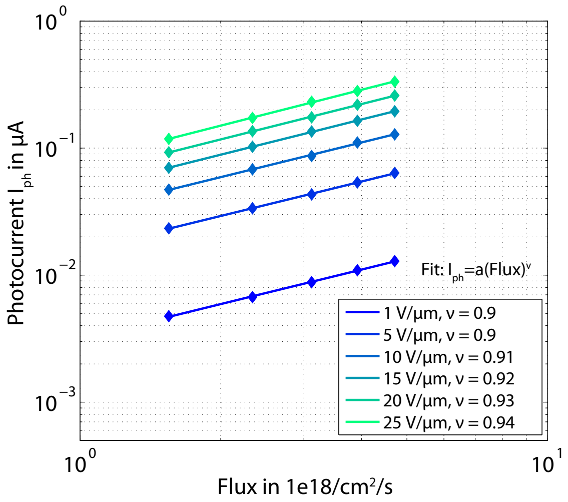

(c)

FIG. 4. Impact of a change of flux on the current-voltage characteristics at 300 K:
a) Current under light I_l for different values of flux (solid lines) as well as dark current I_d (dashed line). b) Photocurrent ($I_{ph} = I_l - I_d$) vs. electric field for different values of flux. c) Photocurrent vs. flux for different values of electric field. A power law was fitted to the data and is indicated by straight lines. The exponent ν describing this power-law is $\nu \sim 0.9$ in rough agreement with calculations from our electronic model, $\nu = 0.99$.

* kaes@physik.rwth-aachen.de
† martin.salinga@physik.rwth-aachen.de

[1] Rütten, M., Kaes, M., Albert, A., Wuttig, M. & Salinga, M. Relation between bandgap and resistance drift in amorphous phase change materials. *Scientific Reports* **5** (2015). URL `http://www.nature.com/articles/srep17362`.

[2] Jost, P. *Charge Transport in Phase-Change Materials*. Ph.d. thesis, RWTH Aachen University (2013).

[3] Oosthoek, J. *et al.* The influence of resistance drift on measurements of the activation energy of conduction for phase-change material in random access memory line cells. *Journal of Applied Physics* **112**, 084506 (2012).

[4] Le Gallo, M., Kaes, M., Sebastian, A. & Krebs, D. Subthreshold electrical transport in amorphous phase-change materials. *New Journal of Physics* **17**, 093035 (2015). URL `http://stacks.iop.org/1367-2630/17/i=9/a=093035`.

[5] Kaes, M., Le Gallo, M., Sebastian, A., Salinga, M. & Krebs, D. High-field electrical transport in amorphous phase-change materials. *Journal of Applied Physics* **118** (2015). URL `http://scitation.aip.org/content/aip/journal/jap/118/13/10.1063/1.4932204`.

[6] Luckas, J. *et al.* The influence of a temperature dependent bandgap on the energy scale of modulated photocurrent experiments. *Journal of Applied Physics* **110**, 013719 (2011).

[7] Raty, J. Y. *et al.* Aging mechanisms in amorphous phase-change materials. *Nature Communications* **6**, 7467 (2015). URL `http://publications.rwth-aachen.de/record/483824`.

[8] Mott, N. F. & Davis, E. A. *Electronic processes in non-crystalline materials* (Clarendon press, 1979).

[9] Elliott, S. R. *Physics of amorphous materials* (Longman London; New York, 1983).

[10] Longeaud, C. *et al.* On the density of states of germanium telluride. *Journal of Applied Physics* **112**, 113714 (2012).
